# Heat and Cold Shocks Decrease the Incidence of Diapause in *Trichogramma telengai* Larvae

**DOI:** 10.3390/insects16010054

**Published:** 2025-01-08

**Authors:** Natalia D. Voinovich, Sergey Y. Reznik

**Affiliations:** Zoological Institute, Russian Academy of Sciences, Universitetskaya 1, 199034 St. Petersburg, Russia; nataliavoinovitch@hotmail.com

**Keywords:** diapause, stress, temperature, cold shock, heat shock, *Trichogramma*

## Abstract

Insect diapause and response to thermal stress are similar in the variety of manifestations. However, the influence of thermal shocks on the incidence of insect diapause has not been sufficiently studied. Our laboratory experiments showed that both cold (−10 °C) and heat (43 °C) shocks experienced for at least 20–30 min significantly reduced the incidence of facultative larval winter diapause in the insect egg parasitoid *Trichogramma telengai*. However, the patterns of these responses were substantially different. In particular, the peaks of the sensitivity to diapause-averting effects of heat and of cold shocks fell, correspondingly, on middle-stage (5 days of development at 15 °C) and late-stage (9–11 days of development at 15 °C) larvae. Heat shocks influence the incidence of diapause mostly via the changes in the initial proportions of diapause-destined and non-diapause-destined individuals, whereas the effect of cold shocks is mostly based on differential mortality (i.e., the difference in mortality among treatments of the same experiment) with better survival of non-diapause-destined individuals. These results elucidate the peculiarities of the interaction between stress and diapause, allowing us to specify the methods for *Trichogramma* mass rearing and storage.

## Simple Summary

It is known that thermal shocks can influence insect diapause but these effects have not been sufficiently studied. *Trichogramma* wasps are egg parasitoids widely used for the biological control of lepidopteran pests. Our laboratory experiments showed that both cold (−10 °C) and heat (43 °C) shocks experienced for at least 20–30 min reduced the percentage of diapause in *Trichogramma* larvae. However, the parameters of these effects are quite different. Heat and cold shocks have the strongest diapause-averting effect, correspondingly, on middle-stage (5 days) and late-stage (9–11 days) larvae. Heat shocks influence the incidence of diapause mostly via the changes in the initial proportions of diapause-destined and non-diapause-destined individuals, whereas the effect of cold shocks is mostly based on differential mortality (i.e., the difference in mortality among treatments of the same experiment) with better survival of non-diapause-destined individuals. These results elucidate the peculiarities of the interaction between stress and diapause allowing us to specify the methods for *Trichogramma* mass rearing and storage. In particular, our study suggests that even short-term exposures of larvae to extremely high temperatures could markedly reduce the proportion of diapausing individuals and thereby decrease their suitability for long-term cold storage.

## 1. Introduction

The diapause syndrome includes substantial morphological, physiological, and behavioral changes in an insect, assuring the survival of unfavorable periods [1,2,3,4,5,6]. By this feature, diapause resembles the response to stress caused by adverse environmental conditions. Stress also has a simultaneous impact on various biological parameters [7,8,9,10]. Moreover, the two processes may partly share the same mechanism of inducing heat shock protein (HSP) expression in insects [11,12,13,14,15,16] and other arthropods [17]. Therefore, it can be assumed that the stress response can have an impact on the incidence of diapause. This effect has been found in some insects but it was rarely studied [5,11,12]. The observed impact of stress on the incidence of diapause can be based on two quite different mechanisms: (1) direct effect exerted on the proportions of diapause-destined and non-diapause-destined individuals and (2) indirect effect exerted via differential mortality (i.e., the difference between diapause-destined and non-diapause-destined fractions in the rate of mortality caused by thermal shock). Thus, it seems necessary to conduct a more detailed study aimed at the separation of the two effects.

For this study, we use *Trichogramma telengai* Sor. (Hymenoptera: *Trichogrammatidae*). *Trichogramma* wasps are minute egg parasitoids widely used in the biological control of lepidopteran pests of agriculture and forestry [18]. The regulation of a facultative larval diapause has been thoroughly investigated in several *Trichogramma* species of the temperate climate zone. Low temperature experienced by embryos and larvae was shown to be the most important diapause-inducing factor. Photoperiod and temperature influencing parental generations are the second important factors. Short days and low temperatures experienced by maternal females promote the induction of diapause in their progeny incubated at near-threshold temperatures [19]. In addition, the incidence of diapause may depend on host egg quality, on parasitoid population density, and on some other minor factors [19,20,21]. However, the impact of thermal shocks on diapause incidence has not been investigated in *Trichogramma* species or any insect parasitoid.

Our main working hypothesis was that thermal shocks would have an impact on the incidence of diapause in *T. telengai*. Moreover, we assumed that not only heat but also cold shocks can influence the proportion of diapause. In previous studies by different authors, only heat shocks were used. In addition, our study aimed to test two secondary hypotheses: (1) extreme temperature directly influences the incidence of insect diapause as one of the diapause-regulating environmental factors and (2) extreme temperature influences the proportion of diapause indirectly via differential mortality of diapause-destined and non-diapause-destined individuals. To achieve these aims we investigated (1) the age-related changes in the sensitivity to heat and cold shocks and (2) changes in the diapause-averting effect in relation to the duration of the exposure to extreme temperatures. If the observed differences from untreated control would be at least partly determined by an increase in the number of diapausing or non-diapausing individuals, this would support the first and refute the second hypothesis because it is highly unlikely that extreme temperatures would increase insect survival. A strong positive correlation between the changes in the diapause incidence and mortality and decrease (or at least constancy) in the number of both diapausing and non-diapausing individuals would rather support the second hypothesis. In particular, the coincidence of a decrease in the diapause incidence with an increase in the absolute number of non-diapausing individuals would support the hypothesis about the direct diapause-averting effect. Otherwise, the coincidence of high mortality with a decrease in the diapause incidence would testify to the differential mortality. However, it should be noted that our working hypotheses are not mutually exclusive: it is also possible that the observed impact of temperature extremes on the incidence of *Trichogramma* diapause (if it would be found) would be determined by the interaction of the two above-mentioned mechanisms.

## 2. Materials and Methods

### 2.1. Insects

The present study was performed on the parthenogenetic strain of *T. telengai* originated from individuals collected in the 1990s in the Moscow province of Russia. In the laboratory, this strain was permanently reared on the eggs of the Angoumois grain moth *Sitotroga cerealella* Olivier. The rearing was conducted at the diapause-averting conditions: 20 °C, air humidity of 70–80%, and L:D = 18:6 (hereafter, photophase and scotophase in h are indicated).

### 2.2. Age-Related Changes

**Figure 1:**
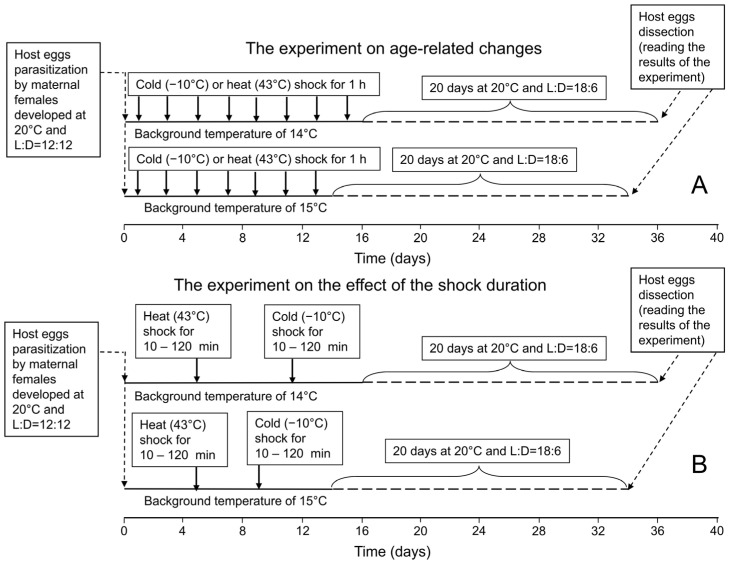
Experimental design. (**A**)—the experiment on the age-related changes; (**B**)—the experiment on the effect of the shock duration. The horizontal lines indicate the development of the experimental individuals: solid lines—at the temperatures of 14° or 15 °C in the dark; dashed lines—at 20 °C and L:D = 18:6. The dashed arrows indicate the beginning and the end of the development of the experimental individuals: left arrow—the parasitization of host eggs by maternal females, right arrow—the dissection of the parasitized host eggs and reading the experimental results. The solid arrows indicate thermal shocks: (**A**)—heat or cold shocks of the same duration (1 h) differed in the larval age at shock (1, 3, 5, 7, 9, 11, 13, or 15 days); (**B**)—heat or cold shocks experienced by larvae of the same age (5 days for heat shocks and 9–11 days for cold shocks) differed in the shock duration (10, 20, 30, 60, or 120 min). Control larvae developed under the same conditions, excluding the thermal shock.

The aim of this experiment (Figure 1A) was to investigate the age-related dynamics of the influence of cold and heat shocks on the incidence of diapause. To start a replicate of this experiment, about 10,000 fresh eggs of the grain moth were glued on a hard paper card by non-toxic polyvinyl acetate (PVA) adhesive and placed for 24 h in a test tube (30 × 100 mm in size) with about 1000 12–24 h old females of the laboratory strain. Then, females were removed and the parasitized grain moth eggs placed in a new test tube were incubated at the temperature of 20 °C (temperature in this and all the other regimes was controlled with the accuracy of 0.3 °C), the relative air humidity of 70–80%, and progeny diapause inducing a short (L:D = 12:12) photoperiod.

The emerged females were individually placed in small (5 × 50 mm) glass test tubes. In each tube, a small paper card with 40–60 fresh eggs of the grain moth was placed for 2 h. Then, females were removed and all the cards with parasitized hosts were mixed into a single pool (cards of a few females that died or escaped during the 2 h parasitization period were excluded). Then, portions of 11–13 cards were randomly selected from the pool, placed in medium-sized test tubes (15 × 100 mm), and distributed among treatments. The experimental treatments differed in the age of larvae subjected to thermal shock. This experiment was conducted with two background temperatures: the larvae were incubated in the dark at either 14 °C or 15 °C. Both temperatures are close to the threshold of diapause induction with the rate of pre-adult development being lower at 14 °C [22]. Each of the two background temperatures was used with two shock temperatures: the cold shock (1 h at −10 °C) or the heat shock (1 h at 43 °C). Shock temperatures and exposure durations were selected based on our pilot experiments. To be subjected to the thermal shock, the test tubes were manually transferred between thermostatic chambers; the temperature change lasted for 8–10 min. Thus, the experiment on age-related changes included 4 combinations of 2 background temperatures with 2 shock temperatures. In addition, each replicate included two controls that were also incubated at 14 or 15 °C but were not subjected to any thermal shock.

At a background temperature of 14 °C, individuals of the 8 experimental treatments were subjected to thermal shocks after 1, 3, 5, 7, 9, 11, 13, or 15 days after parasitization. At a background temperature of 15 °C, individuals of the 7 experimental treatments were subjected to thermal shocks after 1, 3, 5, 7, 9, 11, or 13 days after parasitization. These time periods were selected because for *T. telengai* individuals developed at 14–15 °C, 13–15 days after parasitization are the threshold days to switch to either diapause or direct (non-diapause) development [23]. In 14 and in 16 days after parasitization, all the experimental and control individuals that developed at the background temperatures of 15 and 14 °C, correspondingly were transferred to the temperature of 20 °C and photoperiod of L:D = 18:6 with the aim to speed up pre-adult development and adult emergence of the active (non-diapausing) fraction. In 20 days after the transfer to 20 °C, when adult emergence was completed, all the cards were carefully inspected using a binocular dissecting microscope. On each card, three categories of the blackened host eggs were recorded: (1) diapaused individuals (living larvae), (2) actively developed individuals (empty host chorions with emergence holes), and (3) dead individuals (mostly dead larvae with sporadic dead pupae and pharate adults). Then, the results for all the cards of each replicate of each treatment were summarized. Thus, for each replicate of each treatment of this experiment, the following four parameters were calculated:

- The percentage of blackened host eggs with dead individuals among the total number of blackened host eggs.
- The absolute number of diapausing individuals per one card (i.e., per one female of the maternal generation).
- The absolute number of non-diapausing individuals per one card (i.e., per one female of the maternal generation).
- The percentage of diapause (when this parameter was calculated, dead individuals were excluded).

Each treatment was performed in 8 replicates and each replicate included 10–13 females per treatment.

### 2.3. The Effect of the Shock Duration

The aim of this experiment (Figure 1B) was to study the effects of cold and heat shocks on the proportion of diapausing individuals in relation to the shock duration (the duration of the exposure to extreme temperature). The experiment on the effect of the shock duration was conducted using the same methods as the experiment on age-related changes. It also included 4 combinations of 2 background temperatures (14 °C or 15 °C) with 2 thermal shock temperatures (−10 or 43 °C). Each combination included 6 treatments that differed in the duration of the thermal shock: 0 (control), 10, 20, 30, 60, and 120 min. The cold shock (−10 °C) was applied 9 and 11 days after parasitization for individuals that developed at the background temperatures of 15 and 14 °C, correspondingly. The heat shock (43 °C) was applied 5 days after parasitization for all the individuals. These periods of maximum shock effect on the incidence of diapause were selected based on the results of the previous experiment. Each treatment of the experiment on the effect of the shock duration was performed in 8 replicates and each replicate included 10–13 females per treatment. For each replicate of each treatment of this experiment, the same four parameters as for the previous experiment (see above) were calculated.

### 2.4. Statistical Analysis

The experimental data were not normally distributed and were, therefore, ranked before the statistical analysis. The ranked data for each combination of the background and shock temperatures were analyzed by one-way ANOVA with the larval age at shock (for the first experiment) or the shock duration (for the second experiment) as factors. The four above-mentioned parameters (the percentage of dead individuals, the number of diapausing individuals, the number of non-diapausing individuals, and the percentage of diapausing individuals) were dependent variables. Then, if the influence was statistically significant, treatments were compared with the Tukey HSD test with the same factors and dependent variables. Untransformed data (medians and quartiles) are given in the text and figures. All the calculations were conducted using SYSTAT 10.2 (Systat Software Inc., Richmond, VA, USA).

## 3. Results

### 3.1. Age-Related Dynamics

#### 3.1.1. Cold Shock

**Figure 2:**
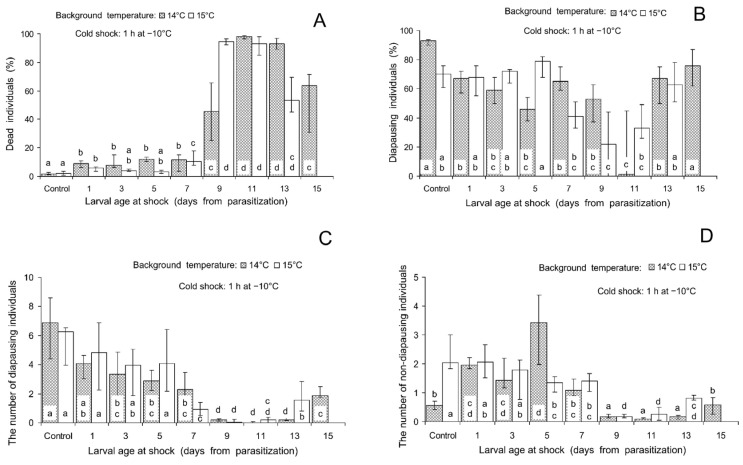
Effect of the cold shock (1 h at −10 °C) on various parameters of *Trichogramma telengai* in relation to background temperature and larval age at shock. (**A**)—the percentage of blackened host eggs with dead individuals, (**B**)—the percentage of diapause, (**C**)—the number of diapausing individuals, and (**D**)—the number of non-diapausing individuals. Medians and quartiles (n = 8) are shown. Bars of the same pattern on the same graph (the data for the same background temperature) with different letters correspond to significantly different values (*p* < 0.05 by Tukey’s HSD test of the ranked data).

The ANOVA showed that the influence of the larval age at shock on mortality and the induction of larval diapause was highly statistically significant for all the combinations of background and shock temperatures (Table 1). In particular, mortality after the cold shock (the temperature of −10 °C experienced during 1 h) was significantly higher than in controls in almost all the experimental treatments. As seen in Figure 2A, mortality in controls was low: 1.6 (1.2–2.7)% and 2.1 (0.7–3.5)% with background temperatures of 14 and 15 °C, correspondingly, but peaked up to 97.9 (96.5–99.0)% and 94.5 (92.4–96.5)% when the shock was applied at the prepupal stage, i.e., 9 and 11 days after parasitization (here and below, treatment medians and quartiles are given). The incidence of diapause in most experimental treatments was lower than in controls (Figure 2B). In controls, the percentage of diapausing individuals was rather high: 93 (90–94)% and 70 (61–76)% with background temperatures of 14 and 15 °C, correspondingly. Minimum incidences of diapause: 1 (0–45)% and 22 (0–44)% were observed when the shock was applied 9–11 days after parasitization. As seen in Figure 2C,D, in these treatments the numbers of both diapausing and non-diapausing living parasitoids were very low. However, as also indicated by a decrease in the percentage of diapause (Figure 2B), non-diapausing individuals survived slightly better. It should be noted that if the shock was applied 13–15 days after parasitization (at the end of the prepupal stage, when pupation began), the decrease in the proportion of diapausing individuals was not significant (Figure 2B) and the percentage of dead individuals was lower than when the shock was applied to 9–11-days-old larvae, although many times higher than in controls (Figure 2A).

#### 3.1.2. Heat Shock

**Figure 3:**
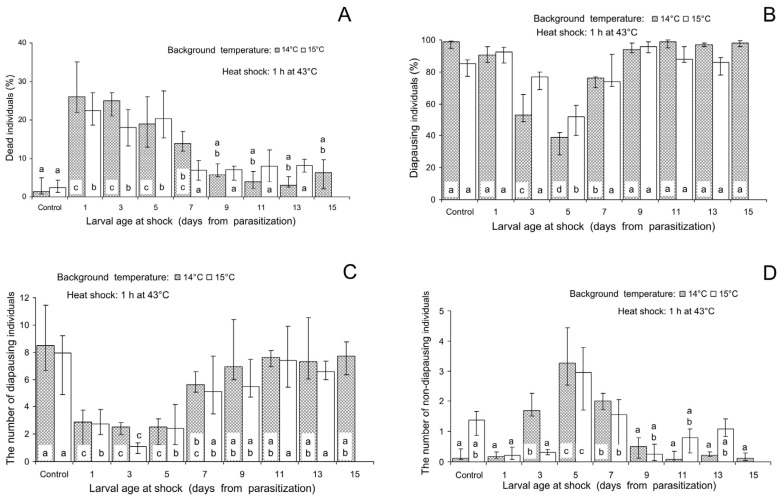
Effect of the heat shock (1 h at 43 °C) on various parameters of *Trichogramma telengai* in relation to the background temperature and larval age at shock. (**A**)—the percentage of blackened host eggs with dead individuals, (**B**)—the percentage of diapause, (**C**)—the number of diapausing individuals, and (**D**)—the number of non-diapausing individuals. Medians and quartiles (n = 8) are shown. Bars of the same pattern on the same graph (the data for the same background temperature) with different letters correspond to significantly different values (*p* < 0.05 by Tukey’s HSD test of the ranked data).

In the heat shock treatments (the temperature of 43 °C experienced during 1 h), mortality in the controls was also low: 1.3 (0.8–4.9)% and 2.4 (1.2–4.4)% with the background temperatures of 14 and 15 °C, correspondingly. The highest mortality, 26.0 (22.1–35.9)% and 22.4 (18.6–27.1)%, was observed when the shock was applied to early- and middle-aged larvae, 1–5 days after parasitization (Figure 3A). The percentage of diapausing controls was high: 98.9 (94.6–99.3)% and 85.3 (77.1–87.5)% with the background temperatures of 14 and 15 °C, correspondingly. The maximum decrease in the proportion of diapausing individuals down to 38.5 (27.5–42.2)% and 52.0 (40.2–58.8)% was observed 5 days after parasitization, about the middle of the larval stage (Figure 3B). This effect was determined not only by a marked decrease in the mean number of diapausing larvae (Figure 3C) but also by a substantial increase in the number of non-diapausing individuals (Figure 3D). For example, when the heat shock was applied to 5-days-old larvae at the background of the temperature of 14 °C, the number of diapausing individuals per maternal female decreased from 8.5 (6.7–11.5) to 2.5 (1.2–3.1), whereas the number of non-diapausing individuals (emerged adults) increased from 0.1 (0.0–0.4) to 3.3 (2.5–4.4) per female.

### 3.2. The Effect of the Shock Duration

#### 3.2.1. Cold Shock

**Figure 4:**
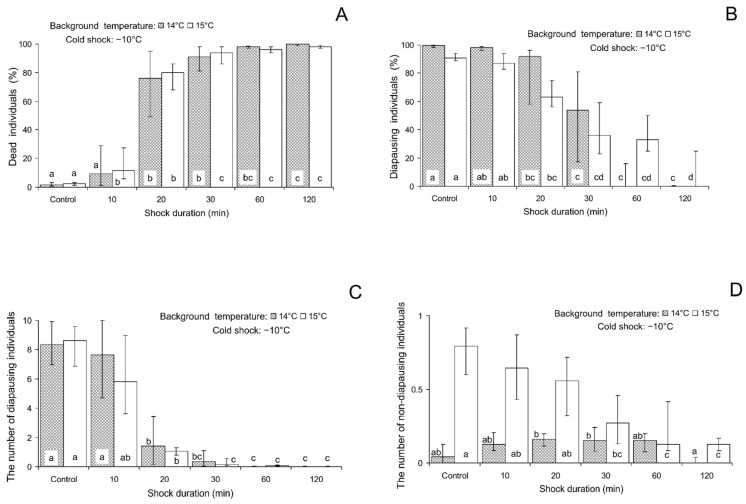
Effect of the cold shock (−10 °C) on various parameters of *Trichogramma telengai* in relation to the background temperature and shock duration. (**A**)—the percentage of blackened host eggs with dead individuals, (**B**)—the percentage of diapause, (**C**)—the number of diapausing individuals, and (**D**)—the number of non-diapausing individuals. Medians and quartiles (n = 8) are shown. Bars of the same pattern on the same graph (the data for the same background temperature) with different letters correspond to significantly different values (*p* < 0.05 by Tukey’s HSD test of the ranked data).

Shock duration has a highly significant impact on mortality and the induction of larval diapause in this experiment (Table 2). As seen in Figure 4A, the mortality of larvae, pupae, and pharate adults in the controls was very low: 1.5 (0.7–3.1)% with the background temperature of 14 °C and 2.4 (1.3–3.2)% with the background temperature of 15 °C. However, mortality sharply increased with the cold shock duration. The percentage of host eggs with dead parasitoids among the total number of darkened eggs was significantly higher than in the controls already after 10–20 min exposure and approached 100% after 1 h and 2 h cold shocks. The percentage of diapausing individuals was very high in the controls: 99.6 (98.3–100.0)% and 90.8 (88.7–93.7)% which decreased with the duration of exposure with the same threshold between 10 and 20 min. After 1 h and 2 h cold shock, depending on the background temperature, the percentage of diapause was close to zero (Figure 4B). The mean number of diapausing larvae per female showed almost the same response (Figure 4C). When the shock was applied on the background of the temperature of 15 °C, the number of non-diapausing individuals per maternal female gradually decreased with the shock duration with the response threshold of 20–30 min (Figure 4D). On the background of 14 °C (at this temperature, the general inclination to enter diapause was somewhat higher), 20–30 min exposures did not cause any statistically significant changes in the number of non-diapausing individuals (Figure 4D). It should be noted that after 1 h and 2 h cold shock, most individuals died (Figure 4A). Therefore, although the few larvae that survived developed without diapause (Figure 4B–D), these data are much less reliable than those of other treatments.

#### 3.2.2. Heat Shock

**Figure 5:**
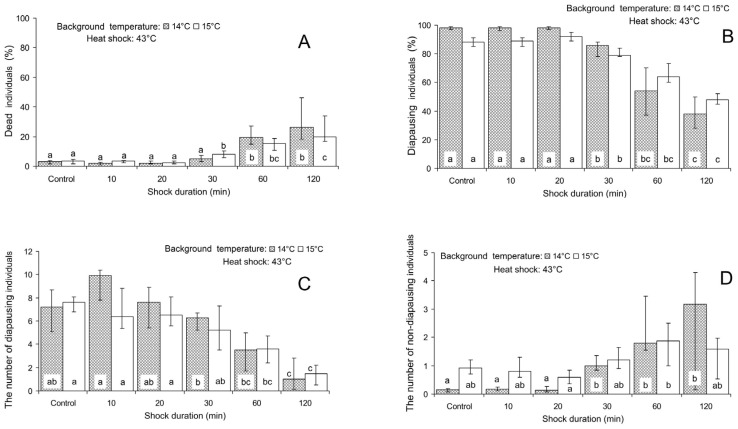
Effect of the heat shock (43 °C) on various parameters of *Trichogramma telengai* in relation to the background temperature and shock duration. (**A**)—the percentage of blackened host eggs with dead individuals, (**B**)—the percentage of diapause, (**C**)—the number of diapausing individuals, and (**D**)—the number of non-diapausing individuals. Medians and quartiles (n = 8) are shown. Bars of the same pattern on the same graph (the data for the same background temperature) with different letters correspond to significantly different values (*p* < 0.05 by Tukey’s HSD test of the ranked data).

The influence of the heat shock experienced during 30 min and longer periods caused a significant increase in *T. telengai* mortality (Figure 5A). With 120 min exposure, mortality reached 26.4 (18.2–46.1) and 20.0 (16.9–33.7)% with the background temperatures of 14 and 15 °C, correspondingly. The incidence of diapause herein decreased with the same response threshold between 20 and 30 min down to 38.4 (27.8–50.0)% and 48.8 (45.8–52.7)% with a shock duration of 120 min (Figure 5B). It should be particularly noted that as well as in the experiment on age-related changes (Figure 3B–D), the last response was caused not only by a decrease in the number of diapausing larvae (Figure 5C) but also by a marked increase in the number of non-diapausing individuals (Figure 5D). In particular, when the 120 min heat shock was applied at the background of the temperature of 14 °C, the median number of diapausing larvae per maternal female decreased from 7.2 (5.1–8.6) to 1.0 (0.2–2.8), whereas the number of non-diapausing individuals (emerged adults) increased from 0.1 (0.1–0.2) to 3.2 (0.2–4.3) per female.

## 4. Discussion

First, we conclude that our main working hypothesis was confirmed: both heat and cold shocks resulted in a substantial decrease in the proportion of diapausing individuals. Effects of thermal stress on the incidence of diapause were observed in some other insects [24,25,26] and arthropods [27]. However, it should be noted that in certain previous studies, the observed changes in the diapause incidence could be caused not by the thermal shock but by the common thermal effect of the induction of diapause. Indeed, it is well known that in many long-day insects, low temperatures induce whereas high temperatures avert facultative winter diapause [1,28,29,30]. In contrast to thermal shocks, these diapause-regulating thermal responses are caused by ‘ecologically relevant’ temperatures that can be experienced in autumn under natural conditions. In addition (also in contrast to thermal shocks), diapause-regulating effects can be caused only by relatively long exposures to different temperatures. In this context, it should be noted that in some previously published experiments, the changes in the proportion of diapausing individuals could be caused rather by diapause-regulating thermal responses than by thermal shocks. For example, this may refer to the study of Colinet et al. [31] when cold exposure (2 °C for 7 days) induced diapause in an aphid parasitoid *Praon volucre* Haliday. Similarly, an increase in the incidence of egg diapause in the band-legged ground cricket, *Dianemobius nigrofasciatus* Matsumura, was caused by 1-day exposure at a temperature of 10 °C [26].

Regarding the ‘true’ thermal shocks, it was shown that the heat shock (2 days at 33 °C or 1 day at 35 °C) experienced by post-fed larvae of flesh fly *Sarcophaga crassipalpis* Macquart markedly decreased the percentage of diapausing individuals [12,24]. Similarly, a decrease in the incidence of diapause in *D. nigrofasciatus* was caused by 1-day exposure at a temperature of 35 °C [26]. It should be noted that as well as in the above-cited studies, in our experiments with *T. telengai*, heat shocks caused a decrease in the proportion of diapausing individuals but not complete prevention of diapause. Practically complete prevention of diapause was observed, for example, in the progeny of *Trichogramma principium* Sug. et Sor and *T. telengai* females of the first post-diapause generation [32,33]. Thus, our data agree with the results obtained by previous investigators. However, the novelty of our study is that (1) we first demonstrated a decrease in the proportion of diapause caused by cold shocks and (2) we first experimentally separated and compared the effects of thermal shocks on the induction of diapause and on the survival of diapause-destined and non-diapause-destined individuals. In addition, in our experiments, the diapause-averting effect was caused by much shorter exposures (not more than 2 h vs. at least 1 day in previous studies).

In this study, heat and cold shocks decreased diapause incidence. The larval ages at shock when diapause incidence decreased differed between heat and cold shocks. In particular, heat shocks caused the maximum decrease in the incidence of diapause when applied to middle-stage (5-days-old) larvae. The maximum response to cold shock was observed in late-stage larvae (9–11 days old depending on the temperature conditions of development). These results suggest that there are critical windows of sensitivity that differ between cold and heat shocks. In our previous study, the maximum sensitivity to the standard diapause-inducing stimulus (3 days at 10 °C) was found in embryos and early larvae (up to 5 days of development at 15 °C), although some weak but statistically significant effect was detected in late larvae up to 12 days of development at 15 °C [23]. Age-related changes in the sensitivity to heat and cold shocks have been found in many insect species. In most studies, the thermal shock effect was evaluated by the changes in the expression level of corresponding genes [34,35] or, as well as in our experiments, by an increase in mortality [36,37,38]. Regarding diapause, Denlinger [24] and Fukumoto et al. [26] noted that the maximum diapause-preventing effects of heat shocks were observed at the time of diapause entry.

In our experiments, the decrease in the incidence of diapause was caused by both heat and cold shocks. In the previous studies, as noted above, only diapause-averting effects of heat shocks were found [12,24,26]. The similarity of stress responses to cold and heat shocks evaluated by the changes in the HSP expression was shown by some authors [15,37]. However, some other studies revealed various differences between stress responses caused by extremely low and extremely high temperatures [38,39,40]. Moreover, although in our study both cold and heat shocks decreased the proportion of diapause, the patterns and the mechanisms of these effects were quite different. As was noted above, peaks of the sensitivity to diapause-averting effects of heat and of cold shocks fall, correspondingly, on middle-stage (5 days old) and late-stage (9–11 days old) larvae.

Our experiments showed that heat and cold shocks differed in the relative importance of the two hypothetical mechanisms of the influence of thermal shocks on the proportion of diapause, which were mentioned in the Introduction: (1) direct effect (the changes in the initial proportions of diapause-destined and non-diapause-destined individuals) and (2) indirect effect (differential mortality of these two fractions). Indeed, the analysis of the age-related changes showed that the decrease in the percentage of diapause caused by the cold shock (Figure 2B) closely coincided with the increase in mortality (Figure 2A). This increase in mortality was observed in both diapausing (Figure 2C) and non-diapausing (Figure 2D) individuals. The peak of mortality caused by the heat shock, on the contrary, was already observed in the very early larval stages (Figure 3A). During this period, the diapause-averting effect was weak or even not yet statistically significant (Figure 3B). The minimum incidence of diapause was recorded when heat shock was experienced by 5-days-old larvae at the very end of the period of high mortality (comp. Figure 3A,B). In addition, in all the cold shock treatments of the experiment on age-related changes, the number of diapausing individuals never exceeded the control values (Figure 2C). The number of non-diapausing parasitoids slightly increased when the cold shock was applied to 1–7-days-old larvae (Figure 2C), long before the period of maximum sensitivity to the diapause-averting effect of the cold shock (Figure 2B). The maximum diapause-averting effect of the heat shock (Figure 3B) coincided both with a significant decrease in the number of diapausing individuals (Figure 3C) and with a marked increase in the number of non-diapausing individuals (Figure 3D). It should also be noted that increases in the number of non-diapausing individuals caused by the cold and heat shocks were observed in 5-days-old larvae. In combination, these data suggest that the diapause-averting effects of both cold and heat shocks may be based on the combination of the direct influence on the initial proportions of diapause-destined and non-diapause-destined larvae and the indirect influence via the mortality of diapause-destined and non-diapause-destined larvae. Herein, the relative role of the direct influence on the induction of diapause by the heat shock is much more important than that of the cold shock. Summarizing, we conclude that both secondary hypotheses of our study are confirmed: thermal shocks influence the incidence of insect diapause both directly (as a diapause-regulating factor) and indirectly (via differential mortality).

In previous studies by various authors, the effects of the shock duration were mostly evaluated by mortality and by the genes’ expression levels [15,35,41,42,43,44,45,46]. It is interesting that these two methods gave somewhat different results. The rate of mortality, as could be expected and as was also shown in our study, monotonously increased with the time of exposure to extreme temperatures up to 100% level [41,42,43,44,45,46]. The synthesis of HSP also increased with the shock duration but this effect was not always monotonous: in some cases, the longest exposures (more than 1 h) resulted in a decrease in the expression level [15,35]. The heat shock effect on the incidence of diapause in *S. crassipalpis* increased when the time of exposure was extended from 1 to 2 days [24]. In our study, the same result was obtained with a much shorter time scale (up to 2 h). Regarding the shock duration effect, it should be noted that the thresholds for the influence on *T. telengai* mortality and on the induction of diapause coincide for both cold (comp. Figure 4A,B) and heat (comp. Figure 4A,B) shocks. Herein, the threshold duration of the cold shock (between 10 and 20 min) is shorter than that of the heat shock (between 20 and 30 min). This suggests that at least with the methods used, the temperature of −10 °C is a stronger stress factor for *T. telengai* larvae than the temperature of 43 °C.

As regards the differential mortality of diapause-destined and non-diapause-destined individuals caused by thermal shocks, it may be explained that diapause has not only benefits but also costs [5,6]. It was repeatedly demonstrated that post-diapause individuals of various insects show lower weight and size, reduced longevity, lower egg load, fecundity, egg hatching rate, etc., than directly developed (non-diapaused) ones, and this effect often increases with diapause duration [47,48,49,50]. On the other hand, some investigations did not confirm this rule or even yielded the opposite results [51,52,53]. It should be noted that all the above-cited studies concerned post-diapause insects. However, the physiological peculiarities of the non-diapause-destined insects of the same species can differ substantially from those of not yet diapaused but diapause-destined individuals [54,55,56,57]. It is also noteworthy that in the latest treatments of the experiment on age-related changes, when the cold shock was applied 13–15 days after parasitization (at the end of the prepupal stage, when pupation began), the rate of mortality decreased (Figure 1) and the proportion of diapausing individuals was not significantly different from that in the controls (Figure 2). Probably, by this time most diapause-destined individuals entered diapause and, as expected, their resistance to environmental extremes increased. Thus, it can be admitted that the preparation for further harsh conditions can somehow reduce the tolerance of diapause-destined individuals to current stressors, and our study provides the first experimental support to this hypothesis.

In combination, the obtained results elucidate the peculiarities of the interaction between stress and diapause. Further studies on this subject would probably reveal the biochemical and molecular mechanisms of this interaction. The influence of ecologically relevant temperatures on the induction of diapause was studied in several *Trichogramma* species [20] including *T. telengai* [22]. It would be interesting to investigate the diapause-inducing effect of the gradient from ecologically relevant to the utmost extreme temperatures. As regards the biological control of insect pests, our data allow us to specify the methods for *Trichogramma* mass rearing and storage. In particular, it should be taken into account that even short-term (1–2 h) exposures of larvae to extremely high temperatures (i.e., those able to induce HSP synthesis and heat stress response) could markedly reduce the proportion of diapausing larvae and thereby decrease their suitability for long-term cold storage.

## Figures and Tables

**Table 1:** Significance of the influence of larval age at thermal shock on various parameters of *Trichogramma telengai* in relation to the shock and background temperatures (the results of one-way ANOVA on the ranked data).

Shock Temperature	Cold Shock (−10 °C)		Heat Shock (43 °C)	
Background temperature	14 °C	15 °C	14 °C	15 °C
Sample size and *df* number	*n* = 72, *df* = 8	*n* = 64, *df* = 7	*n* = 72, *df* = 8	*n* = 64, *df* = 7
Significance of the effect on:				
Percentage of dead individuals	*F* = 55.3, *p* < 0.001	*F* = 52.6, *p* < 0.001	*F* = 18.6, *p* < 0.001	*F* = 22.0, *p* < 0.001
Percentage of diapausing individuals	*F* = 11.0, *p* < 0.001	*F* = 7.2, *p* < 0.001	*F* = 58.8, *p* < 0.001	*F* = 11.2, *p* < 0.001
Number of diapausing individuals	*F* = 44.9, *p* < 0.001	*F* = 27.6, *p* < 0.001	*F* = 18.1, *p* < 0.001	*F* = 13.4, *p* < 0.001
Number of non-diapausing individuals	*F* = 28.4, *p* < 0.001	*F* = 19.3, *p* < 0.001	*F* = 18.5, *p* < 0.001	*F* = 19.3, *p* < 0.001

**Table 2:** Significance of the influence of shock duration on various parameters of *Trichogramma telengai* in relation to the shock and background temperatures (the results of the one-way ANOVA on the ranked data).

Shock Temperature	Cold Shock (−10 °C)		Heat Shock (43 °C)	
Background temperature	14 °C	15 °C	14 °C	15 °C
Sample size and *df* number	*n* = 56, *df* = 5	*n* = 56, *df* = 5	*n* = 56, *df* = 5	*n* = 56, *df* = 5
Significance of the effect on:				
Percentage of dead individuals	*F* = 64.9, *p* < 0.001	*F* = 57.2, *p* < 0.001	*F* = 18.6, *p* < 0.001	*F* = 32.9, *p* < 0.001
Percentage of diapausing individuals	*F* = 15.6, *p* < 0.001	*F* = 26.9, *p* < 0.001	*F* = 58.8, *p* < 0.001	*F* = 23.2, *p* < 0.001
Number of diapausing individuals	*F* = 52.7, *p* < 0.001	*F* = 44.9, *p* < 0.001	*F* = 13.5, *p* < 0.001	*F* = 12.7, *p* < 0.001
Number of non-diapausing individuals	*F* = 3.7, *p* = 0.006	*F* = 11.0, *p* < 0.001	*F* = 11.9, *p* < 0.001	*F* = 3.0, *p* < 0.019

## Data Availability

The original data presented in the study are openly available at Mendeley Data, https://data.mendeley.com/datasets/29j39dghwg/1 (accessed on 6 January 2025).
